# Lower-than-normal myocardial stress and excess hypertrophy from CMR are associated with worsening ventricular contractile performance in hypertrophic cardiomyopathy

**DOI:** 10.1186/1532-429X-18-S1-P3

**Published:** 2016-01-27

**Authors:** Liang Zhong, Xiaodan Zhao, Soo Kng Teo, Haw Chiaw Tang, Yi Su, Ru San Tan

**Affiliations:** 1 National Heart Centre Singapore, Singapore, Singapore; 2 Institute of High Performance Computing, Singapore, Singapore

## Background

Peak systolic myocardial stress was proposed as a stimulus for both adaptive and maladaptive left ventricular (LV) hypertrophy in hypertrophic cardiomyopathy (HCM). We aimed to map segmental myocardial stress and hypertrophy patterns in HCM and correlate them with indices of segmental contractile function.

## Methods

Cine cardiac magnetic resonance (CMR) (Philips Ingenia, 3T) were performed in 19 HCM and 9 normal healthy subjects, from which 3D LV geometric models (each partitioned into 16 segments: 6 basal, 6 mid, 4 distal; apex excluded) were reconstructed. For each segment, the following were determined: (1) wall thickness (h); (2) curvature radius (r), automatically using in-house customized computer algorithm; (3) regional ejection fraction (EF); (4) end-systolic myocardial stress (WS), 0.9 × SBP × r/[2 h × (1+h/2r)], wherein SBP is systolic blood pressure; (5) area strain (AS) (which integrates circumferential, radial, longitudinal deformation and torsion), ln(AS_es_/AS_ed_), wherein AS_ed_ and AS_es_ are end-diastolic and -systolic surface areas, respectively; and (6) stress-corrected AS (Sc-AS), expressed as ratio of AS and AS_predicted, the latter based on a regression equation derived from the normal subjects (AS_predicted = 0.445+3.329/WS). All segments in HCM patients were stratified into two groups: Group 1 with lower-than-normal myocardial stress (myocardial stress <7.6 kN/m^2^) and excess hypertrophy (end-diastolic wall thickness ≥15 mm); Group 2 comprising all other segments. All segments from healthy subjects were considered normal reference.

## Results

Table 1. 14% (43/304) of HCM segments had Group 1 features. Segment regional EF were preserved, but WS, AS and Sc-AS were all decreased in HCM (worse in Group 1 than 2) compared to normal segments.

**Table 1:** Segmental ventricular wall thickness, stress and function in hypertrophic cardiomyopathy and normal subjects

	Normal subjects (144 segments)	HCM Group 2 (261 segments)	HCM Group 1 (43 segments)	ANOVA p value
End-diastolic wall thickness (mm)	6.16 ± 1.36	9.31 ± 2.53#	19.10 ± 3.55#*	<0.001
Wall stress (WS) (× 1000 N/m2)	13.30 ± 5.70	7.46 ± 3.80#	4.23 ± 1.10#*	<0.001
Regional EF (%)	70 ± 10	67 ± 15	64 ± 12	NS
Area strain (AS) (%)	74 ± 18	68 ± 26#	49 ± 12#*	<0.001
Stress-corrected area strain (Sc-AS) (%)	100 ± 20	70 ± 23#	38 ± 10#*	<0.001

# HCM Group 2 and HCM Group 1 vs normal subjects; * HCM Group 1 vs HCM Group 2

## Conclusions

Lower-than-normal myocardial stress and excess hypertrophy is associated with worse segmental ventricular contractile performance.

